# Synthesis and evaluation of highly releasable and structurally stable antibody-SN-38-conjugates

**DOI:** 10.1080/10717544.2021.2008053

**Published:** 2021-12-13

**Authors:** Lianqi Liu, Fei Xie, Dian Xiao, Xin Xu, Zheng Su, Yanming Wang, Shiyong Fan, Xinbo Zhou, Song Li

**Affiliations:** a National Engineering Research Center for the Emergency Drug, Beijing Institute of Pharmacology and Toxicology, Beijing, China; b School of Pharmaceutical Engineering, Shenyang Pharmaceutical University, Shenyang, China

**Keywords:** Antibody-drug conjugates, SN-38, CTSB linkers, ether bond, high DAR values

## Abstract

Camptothecins, traditional chemotherapy drugs, have been clinically used in antibody-drug conjugates (ADCs), which refreshes the recognition that ADCs preferably incorporate highly potent payloads. However, SN-38, active metabolite of irinotecan from camptothecins, tended to be incorporated into ADCs with an unstable acid sensitive bond, not with the widely used Cathepsin B (CTSB) sensitive bond, which may pose the risk of off-target. Herein, we reported a novel strategy to construct highly releasable and structurally stable SN-38-conjugates, in which CTSB linkers directly connected to the 10-OH group through ether bond, not to the common 20-OH group of lactones of SN-38. In this paper, rapid release of SN-38 was skillfully demonstrated by utilizing the fluorescence properties of SN-38. The SN-38-ether-ADC displayed highly stable serum stability with the half-life over 10 days. Moreover, the drug-antibody-ratio (DAR) of ADC could be elevated to 7.1 through the introduction of polyethylene glycol (PEG) moieties without aggregation. The optimized ADC exhibited potent *in vitro* activities up to 5.5 nM, comparable to SN-38. Moreover, this ADC group significantly delayed tumor growth *in vivo*. In conclusion, the novel strategy has the potential to promote the development of SN38-ADCs and enrich the conjugation approaches for hydroxyl-bearing payloads.

## 1. Introduction

Antibody-drug conjugates (ADCs), comprised of tumor‐targeted monoclonal antibodies, potent payloads, and stable linkers, have become one of the most promising fields in cancer therapy (Sievers & Senter, 2013; Abdollahpour-Alitappeh et al., 2019). At present, ten ADCs have been approved and approximately eighty ADCs are currently in clinical trials (Coats et al., 2019; Hafeez et al., 2020). It is generally accepted that payloads with subnanomolar half-maximal inhibitory concentrations (IC_50_ values) have the potential to be incorporated into ADC design (Doronina et al., 2003; Chau et al., 2019; Nakada et al., 2019). Recently, successful applications of camptothecin analogues with nanomolar cytotoxic activity in this field shattered perceptions about ADC design and attracted attentions to this class of toxins. At present, two camptothecin-based ADCs have been approved, DS-8201a (Ogitani et al., 2016), IMMU-132 (Goldenberg et al., 2015), and three others, IMMU-130(Dong et al., 2019), IMMU-140 (Cardillo et al., 2018) and U3-1402 (Yonesaka et al., 2019), are currently in the development phase. For example, IMMU-132, employing the active metabolite of irinotecan SN-38 as the payload, exhibited significant and extensive antitumour effects clinically for metastatic triple-negative breast cancer and urothelial carcinoma (Schreiber et al., 2021; Seligson et al., 2021; Tagawa et al., 2021).

However, of note that there were relatively few reports about SN-38 conjugates. Several scientific issues around two connection sites of SN-38 still need to be addressed. These ADCs adopting 20-OH group of SN-38 as connection site tended to show the instability in serum while others choosing 10-OH group could not release SN-38 in tumor cells timely. For example, IMMU-132 adopted the 20-OH group of SN-38 as the connection site with an acid-sensitive carbonate-based linker, which enhanced the stability of lactone to a great extent (Goldenberg et al., 2015). However, the ester bond connection strategy tended to cause instability of IMMU-132 (t_1/2_ = 23.98 h in human serum) and may pose the risk of off-target. Several attempts have been done for the improvement of the stability of SN-38 based ADCs. For example, CL2E-SN-38 adopted dipeptide-linker, such as valine-citrulline (VC), to connect 10-OH group of SN-38 through two spacers, p-aminobenzyloxycarbonyl (PAB) and ethylenediamine, which exhibited relatively stable in serum (Govindan et al., 2013). However, the extra incorporated ethylenediamine spacer may result in a relatively slow-release rate of SN-38 (Sharkey et al., 2012; Govindan et al., 2013), which may further affect the potency of its conjugates (Bargh et al., 2020; Dal Corso et al., 2020). Additionally, linkers sensitive to the β-glucuronidase in lysosomes have also been described in SN-38 based ADCs (Lau et al., 2018). However, the stereochemical complexity and potential overdependence on one specific enzyme of these linkers, as well as the unexplained low maximum tolerated dose of its conjugates may hinder its further clinical development (Burke et al., 2009; Bargh et al., 2019). So, these SN-38 ADCs still required for further improvements.

**Figure 1.**
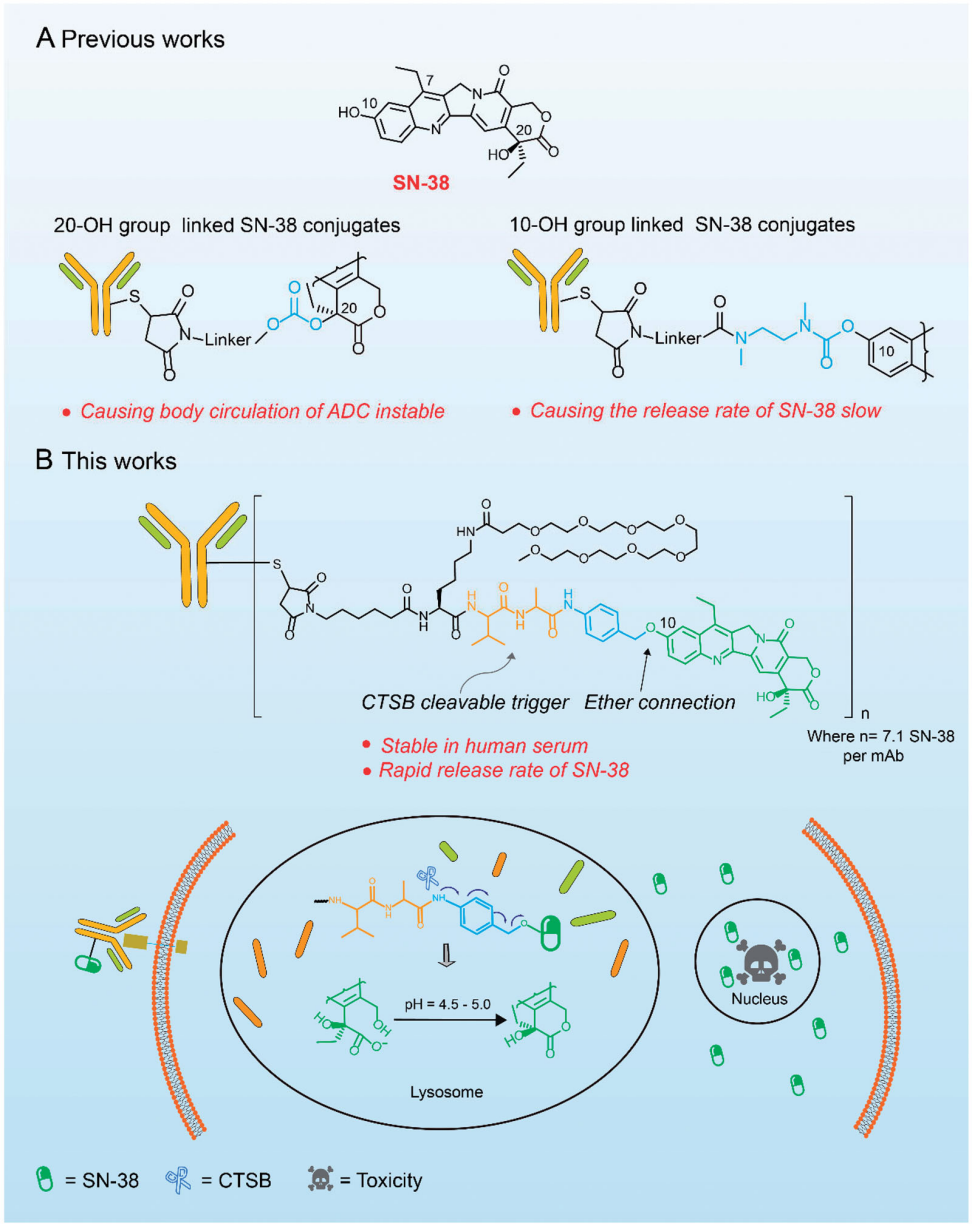
Structures and action mechanisms of SN-38 based ADCs. (A) The major problems facing the SN-38 ADCs with different connection sites. (B) The structure of the constructed ADC Mil40-11 and its mechanism of releasing SN-38 in lysosomes and playing antitumour effects.

In this paper, we proposed a novel strategy to construct highly releasable and structurally stable SN-38-conjugates. The widely used Cathepsin B (CTSB) sensitive linker directly connect to the 10-OH group of SN-38 through ether bond. The ingenuity of this strategy lies in the first verification of dipeptide-phenolic ether fragment releasing payloads rapidly. Meanwhile, the acidic environment of lysosomes (pH = 4.5–5.0) enables the lactone of SN-38 to transform from an open carboxylate, a less active form, into a closed form, thereby maintaining its strong cytotoxicity (Lau et al., 2018). In conclusion, this novel construction strategy of SN-38-based ADCs has the potential to promote the development of SN-38 in ADCs and provide another choice for hydroxyl-group bearing payloads (Figure 1).

## 2. Materials and methods

### 2.1. Materials

All chemical reagents were purchased from commercial sources (Aladdin, Acros, Alfa etc) and were used as received without any further purification. The anti-Her2 antibody Mil40 (a biosimilar of Herceptin®) was purchased from Hisun Pharmaceutical Co., Ltd. (Taizhou, China). Flash column chromatography was used for compound purification. ^1^H NMR spectra of compounds synthesized were recorded at 400 MHz on JEM-ECA-400 magnetic resonance spectrometer using deuterated solvents with tetramethylsilane (TMS) as an internal standard. Chemical shifts were reported in ppm and coupling constants (*J*) in Hz. Mass spectra were recorded with API 3000 mass spectrometer. CTSB from human liver was purchased from Sigma and CTSB inhibitor E-64 from MedChemExpress. The fluorescence emission studies were performed on Microplate reader. All cells were purchased from American Type Culture Collection. Human ovarian carcinoma cell line SKOV-3, breast carcinoma cell line MCF-7 and MDA-MB-231 were cultured in high-glucose DMEM supplemented with 10% FBS, 0.1% penicillin-streptomycin and human breast carcinoma cell line Herceptin-resistant BT474 HerDR was cultured in RPMI-1640 medium containing 10% FBS, 0.1% penicillin-streptomycin.

### 2.2. Synthesis of compounds

#### 2.2.1. Key intermediate compound 4

To the solution of compound 1 (1 g, 2.54 mmol) in anhydrous *N, N*-Dimethylformamide (DMF) (20 mL) under an ice bath was added slowly phosphorus tribromide (680 mg, 2.54 mmol) in anhydrous dichloromethane (30 mL). After 3 h, the reaction was completed and then the solution was transferred into ice water (100 mL). The dichloromethane layer was collected through multiple extractions and dried by anhydrous sodium sulfate overnight. After evaporation of the solvent under vacuum, the crude solid product compound 2 without being purified was obtained (815 mg).

To the solution of compound 2 (800 mg, 1.75 mmol) and SN-38 (689 mg, 1.75 mmol) in anhydrous DMF (25 mL) was added cesium carbonate (573 mg, 1.75 mmol). After being stirred in room temperature for 10 h, the reaction was transferred into the refrigerator for 1 h to precipitate cesium carbonate. After filtration of insoluble solid materials, the crude product was obtained by evaporation of the solution under vacuum and then purified through a silica gel column chromatography using methanol in dichloromethane as an eluent to yield compound 3 (862 mg, 64%). ^1^H NMR (400 MHz, DMSO-d_6_) *δ* 10.06 (s, 1H), 8.03 (d, *J* = 9.2 Hz, 2H), 7.60 (d, *J* = 8.6 Hz, 2H), 7.55 (d, *J* = 2.6 Hz, 1H), 7.51 (dd, *J* = 9.2, 2.7 Hz, 1H), 7.46 (d, *J* = 8.6 Hz, 2H), 7.23 (s, 1H), 6.70 (d, *J* = 8.6 Hz, 1H), 6.48 (s, 1H), 5.39 (s, 2H), 5.25 (s, 4H), 4.42–4.37 (m, 1H), 3.83–3.77 (m, 1H), 3.13 (s, 2H), 1.91 (dd, *J =* 13.5, 6.5 Hz, 1H), 1.86–1.78 (m, 2H), 1.34 (s, 9H), 1.27 (d, *J* = 7.1 Hz, 3H), 1.23 (t, *J* = 7.6 Hz, 3H), 0.84 (t, *J* = 7.2 Hz, 6H), 0.78 (d, *J* = 6.7 Hz, 3H). ESI m/z calculated for C_42_H_49_N_5_O_9_ 767.35, found 768.31.

Compound 3 (862 mg, 1.12 mmol) was dissolved in anhydrous dichloromethane containing 20% trifluoroacetic acid. After being stirred in room temperature 15 min, the crude product was obtained by evaporation of the solution under vacuum and then purified through a silica gel column chromatography using methanol (2%) in dichloromethane as an eluent to yield compound 4 (602 mg, 80.3%) as the light yellow solid. ^1^H NMR (400 MHz, DMSO-d_6_) *δ* 10.24 (s, 1H), 8.75 (d, *J* = 7.0 Hz, 1H), 8.08 (d, *J* = 9.2 Hz, 2H), 7.64 (d, *J* = 8.7 Hz, 2H), 7.59 (d, *J* = 2.6 Hz, 1H), 7.55 (dd, *J* = 9.2, 2.6 Hz, 1H), 7.51 (d, *J* = 8.7 Hz, 2H), 7.27 (s, 1H), 6.53 (s, 1H), 5.43 (s, 2H), 5.30 (s, 4H), 4.54–4.49 (m, 1H), 3.63 (d, *J* = 5.8 Hz, 1H), 3.10 (q, *J* = 7.3 Hz, 1H), 2.08 (dd, *J* = 13.4, 6.7 Hz, 1H), 1.91–1.81 (m, 2H), 1.36 (d, *J* = 7.1 Hz, 3H), 1.27 (t, *J* = 7.6 Hz, 3H), 1.18 (t, *J* = 7.3 Hz, 2H), 0.95 (dd, *J* = 6.9, 3.1 Hz, 6H), 0.88 (t, *J* = 7.3 Hz, 3H). ESI m/z calculated for C_37_H_41_N_5_O_7_ 667.30, found 668.25.

#### 2.2.2. Compound 5

To the solution of compound 4(100 mg, 0.15 mmol) in anhydrous DMF (15 mL) was added 6-Maleimidohexanoic acid N-hydroxysuccinimide ester (55.38 mg, 0.18 mmol) and N, N-Diisopropylethylamine (26 uL, 0.165 mmol). The reaction was stirred overnight under nitrogen protection. Next day, after evaporation of the solvent under vacuum, the crude product was purified through a silica gel column chromatography using methanol in dichloromethane from 1% to 2.5% as an eluent to yield compound 5 (98 mg, 76%) as the light yellow solid. ^1^H NMR (400 MHz, DMSO-d_6_) *δ*10.01 (s, 1H), 8.20 (d, *J* = 6.9 Hz, 1H), 8.08 (d, *J* = 9.2 Hz, 1H), 7.85 (d, *J* = 8.6 Hz,1H), 7.65 (d, *J* = 8.6 Hz, 2H), 7.60 (d, *J* = 2.6 Hz, 1H), 7.57–7.53 (m, 1H), 7.49 (d, *J* = 8.6 Hz, 2H), 7.27 (s, 1H), 7.01 (d, *J* = 2.0 Hz, 2H), 6.53 (s, 1H), 5.43 (s, 2H), 5.29 (s, 4H), 4.41–4.36 (m, 1H), 4.16 (d, *J* = 8.6 Hz, 1H), 3.62 (dd, *J* = 6.6, 2.7 Hz, 4H), 3.17–3.11 (m, 6H), 2.14 (dt, *J* = 21.9, 7.3 Hz, 2H), 1.96 (dd, *J* = 13.6, 6.6 Hz, 1H), 1.93–1.77 (m, 2H), 1.50–1.44 (m, 3H), 1.23 (s, 3H), 0.92–0.84 (m, 6H), 0.82 (d, *J* = 6.8 Hz, 3H). ESI m/z calculated for C_47_H_52_N_6_O_10_ 860.37, found 861.30.

#### 2.2.3. Compound 6

To the solution of compound 4(100 mg, 0.15 mmol) in anhydrousDMF (15 mL) was added N-Hydroxysuccinimidyl-(polyethylene glycol) 2-maleimide (76.5 mg, 0.18 mmol) and N, N-Diisopropylethylamine (26 uL, 0.165 mmol). The reaction was stirred overnight under nitrogen protection. Next day, after evaporation of the solvent under vacuum, the crude product was purified through a silica gel column chromatography using methanol in dichloromethane from 1% to 2.5% as an eluent to yield compound 6 (101 mg, 68.8%) as the light yellow solid. ^1^H NMR (400 MHz, DMSO-d_6_) *δ* 10.01 (s, 1H), 8.23 (d, *J* = 7.1 Hz, 1H), 8.06 (dd, *J* = 14.3, 7.3 Hz, 2H), 7.92 (d, *J* = 8.6 Hz, 1H), 7.65 (d, *J* = 8.6 Hz, 2H), 7.59 (d, *J* = 2.5 Hz, 1H), 7.55 (dd, *J* = 9.2, 2.5 Hz, 1H), 7.50 (d, *J* = 8.6 Hz, 2H), 7.26 (s, 1H), 7.00 (s, 2H), 6.53 (s, 1H), 5.43 (s, 2H), 5.29 (s, 4H), 4.42–4.37 (m, 1H), 4.25–4.19 (m, 1H), 3.62–3.56 (m, 4H), 3.47 (s, 2H), 3.34 (s, 2H), 3.14 (d, *J* = 3.4 Hz, 2H), 2.47–2.38 (m, 2H), 2.33 (t, *J* = 7.3 Hz, 2H), 1.98–1.93 (m, 1H), 1.90–1.82 (m, 2H), 1.31 (d, *J* = 7.1 Hz, 2H), 1.29–1.26 (m, 6H), 1.25 (d, *J* = 1.4 Hz, 2H), 0.90–0.85 (m, 6H), 0.83 (d, *J* = 6.8 Hz, 3H). ESI m/z calculated for C_51_H_59_N_7_O_13_ 977.42, found 978.42.

#### 2.2.4. Compound 7

To the solution of compound 4(100 mg, 0.15 mmol) in anhydrous DMF (15 mL) was added N-Hydroxysuccinimidyl-(polyethylene glycol) 4-maleimide (92.37 mg, 0.18 mmol) and N, N-Diisopropylethylamine (26 uL, 0.165 mmol). The reaction was stirred overnight under nitrogen protection. Next day, after evaporation of the solvent under vacuum, the crude product was purified through a silica gel column chromatography using methanol in dichloromethane from 1% to 2.5% as an eluent to yield compound 7 (86 mg, 53.8%) as the light yellow solid. ^1^H NMR (400 MHz, DMSO-d_6_) *δ* 9.99 (s, 1H), 8.22 (d, *J =* 7.1 Hz, 1H), 8.08 (d, *J* = 9.2 Hz, 1H), 8.04 (t, *J* = 5.6 Hz, 1H), 7.91 (d, *J* = 8.5 Hz, 1H), 7.65 (d, *J* = 8.6 Hz, 2H), 7.60 (d, *J* = 2.5 Hz, 1H), 7.55 (dd, *J* = 9.1, 2.5 Hz, 1H), 7.50 (d, *J* = 8.6 Hz, 2H), 7.27 (s, 1H), 7.00 (s, 2H), 6.52 (s, 1H), 5.43 (s, 2H), 5.29 (s, 4H), 4.42–4.37 (m, 1H), 4.24–4.19 (m, 1H), 3.62–3.56 (m, 4H), 3.48 (d, *J* = 5.4 Hz, 10H), 3.18 (d, *J* = 9.4 Hz, 2H), 3.14 (d, *J* = 5.7 Hz, 2H), 2.47–2.38 (m, 2H), 2.35–2.30 (m, 2H), 1.96 (dd, *J* = 13.6, 6.8 Hz, 1H), 1.91–1.82 (m, 2H), 1.31 (d, *J* = 7.1 Hz, 2H), 1.28–1.24 (m, 6H), 1.23 (d, *J* = 1.7 Hz, 2H), 0.91–0.85 (m, 6H), 0.83 (d, *J* = 6.8 Hz, 3H). ESI m/z calculated for C_55_H_67_N_7_O_15_ 1065.47, found 1066.50.

#### 2.2.5. Compound 8

To the solution of compound 4(100 mg, 0.15 mmol) in anhydrous DMF (15 mL) was added N-Hydroxysuccinimidyl-(polyethylene glycol) 8-maleimide (123.72 mg, 0.18 mmol) and N, N-Diisopropylethylamine (26 uL, 0.165 mmol). The reaction was stirred overnight under nitrogen protection. Next day, after evaporation of the solvent under vacuum, the crude product was purified through a silica gel column chromatography using methanol in dichloromethane from 1% to 2.2% as an eluent to yield compound 8 (117 mg, 62.9%) as the light yellow solid. ^1^H NMR (400 MHz, DMSO-d_6_) *δ* 9.99 (s, 1H), 8.22 (d, *J* = 7.1 Hz, 1H), 8.08 (d, *J* = 9.2 Hz, 1H), 8.04 (t, *J* = 5.3 Hz, 1H), 7.91 (d, *J* = 8.8 Hz, 1H), 7.65 (d, *J* = 8.6 Hz, 2H), 7.60 (d, *J* = 2.6 Hz, 1H), 7.55 (dd, *J* = 9.2, 2.6 Hz, 1H), 7.50 (d, *J* = 8.6 Hz, 2H), 7.27 (s, 1H), 7.01 (d, *J* = 2.6 Hz, 2H), 6.53 (s, 1H), 5.43 (s, 2H), 5.30 (s, 4H), 4.43–4.36 (m, 1H), 4.24–4.18 (m, 1H), 3.61–3.56 (m, 4H), 3.50 (t, *J* = 3.5 Hz, 24H), 3.35 (s, 4H), 3.21–3.12 (m, 4H), 2.44–2.35 (m, 2H), 2.34–2.30 (m, 2H), 1.96 (dd, *J* = 13.5, 6.7 Hz, 1H), 1.90–1.80 (m, 2H), 1.29 (dt, *J* = 27.5, 8.8 Hz, 8H), 0.91–0.78 (m, 9H). ESI m/z calculated for C_63_H_83_N_7_O_19_ 1241.57, found 1242.55.

#### 2.2.6. Compound 11

To the solution of compound 4 (694 mg, 1.04 mmol) in anhydrous DMF (20 mL) was added Fmoc-Lys (PEG_8_)-NHS ester (900 mg, 1.04 mmol) and *N*, *N*-Diisopropylethylamine (148 mg, 1.14 mmol). After being stirred at room temperature for 10 h, the crude product was obtained by evaporation of the solution under vacuum and then purified through flash chromatography using methanol in dichloromethane, ranging from 2% to 7%, to yield compound 9 (923 mg, 62.8%) as the light yellow solid. ^1^H NMR (400 MHz, DMSO-d_6_) *δ* 10.06 (s, 1H), 8.24 (d, *J* = 6.2 Hz, 1H), 8.08 (d, *J* = 9.2 Hz, 1H), 7.88 (d, *J* = 7.3 Hz, 2H), 7.83 (t, *J* = 5.8 Hz, 1H), 7.72 (t, *J* = 6.7 Hz, 3H), 7.64 (d, *J* = 8.5 Hz, 2H), 7.60 (d, *J* = 2.6 Hz, 1H), 7.55 (dd, *J* = 9.0, 2.8 Hz, 2H), 7.49 (d, *J* = 8.6 Hz, 2H), 7.41 (t, *J* = 7.4 Hz, 2H), 7.32 (t, *J* = 7.4 Hz, 2H), 7.27 (s, 1H), 6.53 (s, 1H), 5.43 (s, 2H), 5.29 (s, 4H), 4.42–4.35 (m, 1H), 4.34–4.25 (m, 2H), 4.24–4.18 (m, 2H), 4.03 (td, *J* = 9.3, 5.2 Hz, 1H), 3.57 (t, J = 6.5 Hz, 2H), 3.54–3.43 (m, 23H), 3.41 (dd, *J* = 5.8, 3.3 Hz, 2H), 3.22 (s, 2H), 3.18 (d, *J* = 7.4 Hz, 2H), 3.01 (dt, *J* = 13.8, 6.8 Hz, 2H), 2.29 (t, *J* = 6.5 Hz, 2H), 1.98 (dd, *J* = 13.1, 7.0 Hz, 1H), 1.91–1.82 (m, 2H), 1.57 (dd, *J* = 44.9, 8.7 Hz, 3H), 1.37 (dd, *J* = 18.6, 4.9 Hz, 3H), 1.33–1.26 (m, 6H), 1.24 (d, *J* = 9.6 Hz, 4H), 0.88 (t, *J* = 7.1 Hz, 6H), 0.83 (d, *J* = 6.6 Hz, 3H). ESI m/z calculated for C_76_H_97_N_7_O_19_ 1411.68, found 1412.69.

Compound 9 (900 mg, 0.63 mmol) was dissolved in anhydrous dichloromethane containing 5% piperidine. After being stirred at room temperature for 2 h, the crude product was purified through flash chromatography using methanol in dichloromethane, ranging from 2% to 5%, to yield compound 10 (516 mg, 68.8%) as the light yellow solid. ESI m/z calculated for C_61_H_87_N_7_O_17_ 1189.62, found 1190.64.

Compound 10 (100 mg, 0.08 mmol) and 6-Maleimidohexanoic acid N-hydroxysuccinimide ester (29 mg, 0.08 mmol) were dissolved in anhydrous DMF (20 mL). The solution was stirred at room temperature overnight. After evaporation of the solvent under vacuum, the crude product was obtained by evaporation of the solution under vacuum and then purified through flash chromatography using methanol in dichloromethane, ranging from 2% to 7%, to yield compound 11 (75 mg, 63.5%) as the light yellow solid. ^1^H NMR (400 MHz, DMSO-d_6_) *δ* 10.03 (s, 1H), 8.19 (d, *J* = 7.1 Hz, 1H), 8.08 (d, *J* = 9.2 Hz, 1H), 7.96 (d, *J* = 8.0 Hz, 1H), 7.80 (t, *J* = 5.6 Hz, 1H), 7.70–7.62 (m, 3H), 7.60 (d, *J* = 2.6 Hz, 1H), 7.56 (dd, *J* = 9.2, 2.5 Hz, 1H), 7.49 (d, *J* = 8.6 Hz, 2H), 7.27 (s, 1H), 7.00 (s, 2H), 6.52 (s, 1H), 5.43 (s, 2H), 5.30 (d, *J* = 3.0 Hz, 4H), 4.41–4.35 (m, 1H), 4.24 (dd, *J* = 10.9, 5.9 Hz, 1H), 4.21–4.16 (m, 1H), 3.57 (t, *J* = 6.5 Hz, 2H), 3.48 (d, *J* = 13.3 Hz, 22H), 3.42 (dd, *J* = 5.8, 3.3 Hz, 2H), 3.39–3.33 (m, 4H), 3.23 (s, 2H), 3.00 (dd, J = 15.3, 7.0 Hz, 2H), 2.28 (t, *J* = 6.6 Hz, 2H), 2.15–2.04 (m, 2H), 2.01–1.95 (m, 1H), 1.92–1.80 (m, 2H), 1.61 (s, 2H), 1.47 (dd, *J* = 13.3, 6.6 Hz, 4H), 1.40–1.32 (m, 4H), 1.29 (dd, *J* = 13.5, 7.2 Hz, 6H), 1.23 (s, 4H), 1.19 (d, *J* = 7.0 Hz, 3H), 0.93–0.84 (m, 6H), 0.82 (d, *J* = 6.7 Hz, 3H). ESI m/z calculated for C_71_H_98_N_8_O_20_ 1382.69, found 1405.62.

#### 2.2.7. Compound 20

*tert*-butyl (4-(bromomethyl)phenyl)carbamate (100 mg, 0.35 mmol) and SN-38 (137.5 mg, 0.35 mmol) were dissolved in anhydrous DMF (20 mL). The solution was stirred at room temperature overnight. The crude product was obtained by evaporation of the solution under vacuum and then purified through flash chromatography using methanol in dichloromethane, ranging from 2% to 5%, to yield compound 20 (79 mg, 38%) as the light yellow solid. ^1^H NMR (400 MHz, DMSO-d_6_) *δ* 10.01 (s, 1H), 8.20 (d, *J* = 6.9 Hz, 1H), 8.08 (d, *J* = 9.2 Hz, 1H), 7.85 (d, *J* = 8.6 Hz, 1H), 7.65 (d, *J* = 8.6 Hz, 2H), 7.60 (d, *J* = 2.6 Hz, 1H), 7.57–7.53 (m, 1H), 7.49 (d, *J* = 8.6 Hz, 2H), 7.27 (s, 1H), 7.01 (d, *J* = 2.0 Hz, 2H), 6.53 (s, 1H), 5.43 (s, 2H), 5.29 (s, 4H), 4.41–4.36 (m, 1H), 4.16 (d, *J* = 8.6 Hz, 1H), 3.62 (dd, *J* = 6.6, 2.7 Hz, 4H), 3.17–3.11 (m, 6H), 2.14 (dt, *J* = 21.9, 7.3 Hz, 2H), 1.96 (dd, *J* = 13.6, 6.6 Hz, 1H), 1.93–1.77 (m, 2H), 1.50–1.44 (m, 3H), 1.23 (s, 3H), 0.92–0.84 (m, 6H), 0.82 (d, *J* = 6.8 Hz, 3H). ESI m/z calculated for C_34_H_35_N_3_O_7_ 597.25, found 598.26.

### 2.3. Preparation of Mil40-SN38 conjugates

The storage buffer (20 mM L-Histidine, pH = 5.5) and reaction buffer (20 mM L-Histidine, pH = 7.2–7.5) of Humanized anti-Her2 IgG1 antibodies Mil40 were prepared. Compound 5, 6, 7, 8, 11 was dissolved in DMA as a stock solution, respectively. Mil40 in reaction buffer was treated with 2.4–3.0 mol equivalent TCEP to reduce the interchain disulfide bridges between the heavy-heavy and heavy-light chains for 2 h at 25 °C. To the solution of the reduced Mil40 was added 8 mol equivalent of compound 5, 6, 7, 8, or 11 in DMA. The conjugation reaction was allowed to proceed at 25 °C for 3 h with gentle mixing and the final concentration of Mil40 used in reactions were 5 mg/mL. After completion of conjugation, the reaction was quenched with excess *N*-acetylcysteine (NAC). Conjugates were exchanged into storage buffer using Sephadex-G25S to remove the free NAC-5, 6, 7, 8, or 11 and then were stored at −80 °C before use for analysis and testing.

To further elevate the DAR values of conjugates, Mil40 in reaction buffer was treated with 8 mol equivalent TCEP to reduce the interchain disulfide bridges, then the solution of reduced Mil40 was added 15 mol equivalent of compound 5, or 11 in DMA. The conjugation reaction was allowed to proceed at room temperature for 24 h. Other operations were the same as the mentioned at first.

### 2.4. Quality control

DAR values of ADCs were analyzed by hydrophobic interaction chromatography (HIC), UPLC-Q-TOF-MS and the polymerization degree was determined by size exclusion chromatography. HIC analysis was carried out on a TSK-gel Butyl-NPR column (2.5 μm, 4.6 mm × 3.5 cm, TOSOH Bioscience). Mobile phase A was 25 mM sodium phosphate and 1.8 M ammonium sulfate in water. Mobile phase B was 25 mM sodium phosphate in water containing 20% isopropanol. The gradient was 0% to 100% mobile phase B until 18.00 min, followed by re-equilibration from 18.00–18.01 min with 100% mobile phase A and isocratic step at 100% mobile phase A from 18.01–24.00 min, flow rate was 0.8 mL/min, and the UV detection wavelength was 280 nM. The ADC DAR was determined by calculating from the sum of the weighted ratios of the area under the peak multiplied by corresponding peak drug loading through HIC analysis. UPLC-Q-TOF-MS analysis was measured on Agilent 6550 LC-Q-TOF MS spectrometer with a TSK UP-SW3000 column (4.6 × 150 mm, 2.5 μm). The column was eluted with an isocratic step at 0.2 mL/min with 200 mM ammonium acetate solution. The multiple charged peaks of ADCs were deconvoluted using the Agilent MassHunter Bioconfirm sofware (deconvolution for protein, Agilent technology).

The capacity of Mil40-11 binding to Her2 antigen compared with monoclonal antibody Mil40 was determined by ELISA. The detailed protocol of measuring the affinity of Mil40-11 to Her2 antigen was reported previously (Li et al., 2021; Xiao et al., 2021).

### 2.5. SN-38 released from phenolic ether

Released SN-38 was monitored by HPLC after the removal of Boc fragment of compound 20. Taking advantage of the self-fluorescence of SN-38, the process of releasing payload from phenolic ether was studied. NAC-6, NAC-11, or NAC-21 was prepared by the reaction of compound 6, 11, or 21 with excessive NAC. The stock solution was prepared in triple distilled water containing DMSO (50%, v/v). Stock solution of E-64 of CTSB inhibitor was dissolved in triple distilled water. Stock solutions of CTSB with different concentrations were prepared in CTSB activity buffer (50 mM sodium acetate, 100 mM NaCl, 8 mM L-cysteine, 1 mM EDTA, pH 5.0). Fluorescence responses of NAC-21 (5 μM) in CTSB activity buffer containing DMSO (10%, v/v) to CTSB (3 UN/mL) were recorded after incubation at 37 °C at different time points. Kinetics of fluorescence response of NAC-21 (5 μM) was recorded in the presence of varying concentrations of CTSB. The role of CTSB in releasing SN-38 was determined in the presence of CTSB inhibitor E-64.

### 2.6. Serum stability

To determine the stability of Mil40-11 in human plasma, SN-38 and 10-hydroxycamptothecin as an internal standard were diluted into 2 × diluted human plasma to prepare the standard sample, and Mil40-11 sample preparation was the same as standards. Both samples were incubated at 37 °C for a period of 10 days. The concentration of total free SN-38 released from the Mil40-11 conjugates samples was determined at various time-points according to the established standards using LC-MS. Chromatographic experiments were performed using a liquid chromatographic system consisting of Waters Alliance HPLC system equipped with a Sample Manager, a PDA detector and a QDa detector which is a compact single quad mass detector equipped with the electrospray ionization (ESI) interface (Waters, Czech Republic). Mobile phase A was 0.1% formic acid in water and mobile phase B was 0.1% formic acid in acetonitrile. A gradient elution was performed with 20% mobile phase B for 2 min, a linear increase to 100% mobile phase B until 3.0 min, followed by isocratic step at 100% mobile phase B from 3–5 min and re-equilibration from 5.1–8 min with 20% mobile phase B. The flow rate was set to 0.8 mL/min.

### 2.7. In vitro antitumor activities of Mil40-SN-38 conjugates

The growth inhibition of the SN-38, Mil40, and a series of antibody-drug conjugates mentioned were determined by cell viability assay. Briefly, BT-474 HerDR (8.0 × 10^3^ cells/well), SKOV-3 (2.5 × 10^3^ cells/well), MCF-7 (3.0 × 10^3^ cells/well), MDA-MB-231 (1.5 × 10^3^ cells/well) were seeded in 100 μL medium in 96-well plates and further incubated for 24 h. Cells were treated with different concentrations of SN-38, Mil40, and a series of antibody-drug conjugates, respectively and incubated at 37 °C for 6 days except BT474 HerDR for 9 days and SKOV-3 for 10 days. Cell viability assay was measured using Cell Titer-Glo luminescent cell ability kit. IC_50_ values were calculated with a 4-variable curve analysis using Origin 2018 software.

### 2.8. Flow cytometric analysis

Flow cytometry was applied to determine the targeting of Mil40-11 against SKOV-3 cells and MDA-MB-231 cells. SKOV-3 cells (5 × 10^5^ cells/sample) and MDA-MB-231 cells (5 × 10^5^ cells/sample) were harvested under the log phase of growth and suspended in FACS clean solution (PBS containing 2% fetal bovine serum). These cells were treated with Mil40-11 and Mil40 diluted in 100 μL PBS at the concentration of 5 ug/mL, or PBS as blank control at 4 °C for 30 min, respectively. Then, the cells were washed twice with FACS clean solution and incubated with PE-conjugated anti-human IgG at 4 °C for 30 min. Then, after being washed three times with FACS clean solution, cells were finally detected by flowed cytometry (BD Biosciences). The ratio of mean fluorescence intensity (rMFI) was used to reflect the targeting ability.

Meanwhile, SKOV-3 cells were harvested and suspended in FACS clean solution. Experimental and control groups were all treated with Mil40, or Mil40-11 at 4 °C for 1 h. After being washed twice with prechilled FACS clean solution, experimental groups were incubated at 37 °C for 45 min, or 8 h, while the control groups were still incubated at 4 °C for the set time points. All samples were washed three times with prechilled FACS clean solution when reaching the time points, and then incubated with PE-conjugated anti-human IgG at 4 °C for 30 min. Flow cytometry was used to determine the rMFI, and internalization percentage was calculated with rMFI by the following formula: internalization rates (%) = (100 - (rMFI of experimental groups/rMFI of control groups)) × 100%.

### 2.9. In vitro cell imaging

SKOV-3 cells were seeded on chamber and incubated for 24 h at 37 °C under 5% CO_2_ and 95% air. After removal of medium, cells were treated with FITC labeled Mil40, or FITC labeled Mil40-11 in medium at 4 °C for 30 min, or 37 °C for 24 h. Groups incubated at 4 °C were for observation of the affinity of Mil40, or Mil40-11 to SKOV-3 cells and groups incubated at 37 °C for the observation of internalization of Mil40, or Mil40-11 into SKOV-3 cells. When reaching the time points, cells were washed with PBS and stained with Lyso-tracker Red at lysosome and DAPI at nucleus. After being washed three times with PBS, fluorescent images were taken under a fluorescent microscope.

### 2.10. In vivo antitumor activity in human ovarian xenograft model

All studies were conducted in compliance with accepted standards for the care and use of laboratory animals. Three BALB/c-nude mice (6–8 weeks old) were injected subcutaneously with SKOV-3 cells (1 × 10^7^ cells). When the volume of tumors reached approximate 500 mm^3^, the mice were killed and tumors were taken out, followed with the removal of envelope and necrotic tissues. The weighed 10 grams tumors were cut into a paste with a scalpel on ice and then well-mixed with 10 mL RPMI-1640 medium into tumor tissue suspension. Each mouse was subcutaneously inoculated with 0.2 mL tumor suspension. When the tumors reached a volume of approximately 100 mm^3^, nude mice were randomly assigned to seven treatment groups (*n* = 7–8/group):control group (L- His buffer), three Mil40-11(DAR = 7.1) treatment groups (5 mg/kg,10 mg/kg, and 20 mg/kg), two Mil40 treatment groups (10 mg/kg and 20 mg/kg) and the drug combination group (20 mg/kg Mil40-11 + 0.66 mg/kg irinotecan). The seven treatment groups (250 μL) were injected by tail vein into mice at 0, 3, 7, 10 days. Subcutaneous tumors were measured using caliper every three or four days, and the tumor volume calculated as 1/2 × length × width × width (mm). Animals were terminated when tumors grew to 2.5 cm^3^. Mice body weight was also monitored as a marker of toxicity. At the end of study, tumors of mice were collected and further analyzed by H&E staining.

## 3. Results and discussion

### 3.1. Chemistry

**Scheme 1:**
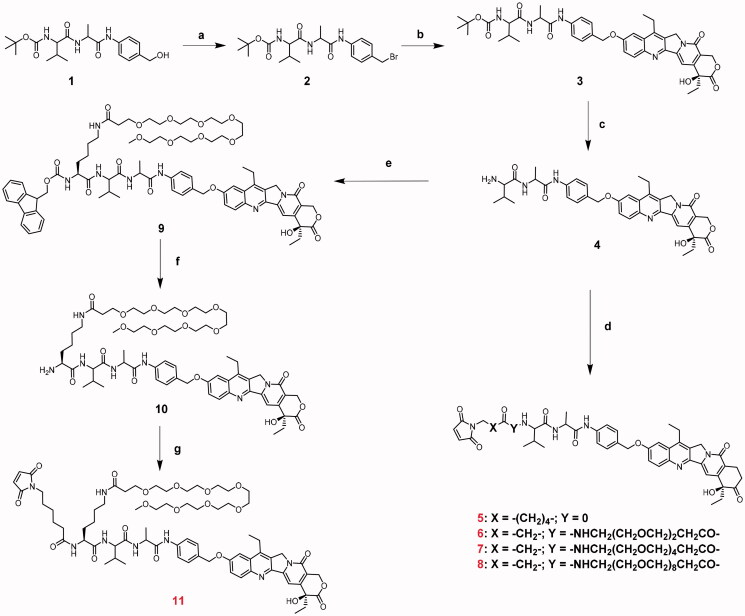
Synthesis of the SN-38-based linkers. Reagents and conditions: All reactions were performed at room temperature unless otherwise noted. (a) Phosphorus tribromide, dimethylformamide, dichloromethane, 3 h, 0 °C. (b) SN-38, cesium carbonate, dimethylformamide, 10 h. (c) trifluoroacetic acid, dichloromethane, 15 min. (d) the specific maleimides are all listed in the Experimental Section, DIPEA, dimethylformamide, overnight. (e) Fmoc-Lys (PEG_8_)-NHS ester, DIPEA, dimethylformamide, 10 h. (f) piperidine, dichloromethane, 2 h. (g) 6-Maleimidohexanoic acid N-hydroxysuccinimide ester, DIPEA, dimethylformamide, overnight.

The synthetic routes of Linker-SN-38, including compounds 5, 6, 7, 8, and 11, are shown in Scheme 1. The key intermediate compound 4 was obtained first in the following steps. Compound 1 was converted into compound 2 through bromination reactions. Because of the steric hindrance of α-hydroxy lactone, the hydroxy group of SN-38 does not require protection when preparing compound 3, which was obtained through a phenolic ether connection (Lau et al., 2018). The key intermediate compound 4 was finally obtained after removal of the Boc protection group and then reacted with maleimides containing different lengths of polyethylene glycol (PEG) to yield target compounds 5, 6, 7, and 8. Overall ADC incorporating the pendant PEG linker may exhibit the better hydrophilic properties than that of straight PEG linker. (Lyon et al., 2015; Tedeschini et al., 2021; Xiao et al., 2021) Compound 4 reacted with activated Fmoc-Lys (PEG_8_)-OH to provide compound 9. Fmoc deprotection followed by maleimide incorporation yielded the desired compound 11 containing the pendant PEG moiety. Detailed synthetic procedures are shown in the Experimental Section.

### 3.2. Antibody conjugation

Compounds 5, 6, 7, 8, and 11 were conjugated to the humanized monoclonal antibody Mil40. The well-studied Mil40, marketed biosimilar of Herceptin, targets the Her2 antigen. The intermolecular disulfide bonds of Mil40 was reduced with TCEP to expose the sulfhydryl groups, followed by in situ conjugation with maleimide-containing compounds 5, 6, 7, 8, or 11. The conjugation was terminated with excess of NAC. The DAR of prepared Mil40-5,6,7,8 ADCs were determined by HIC. To elevate the DAR values of ADCs, Mil40 was reduced with 8-fold molar TCEP for 2 h.

### 3.3. *In vitro* SN-38 release

**Figure 2.**
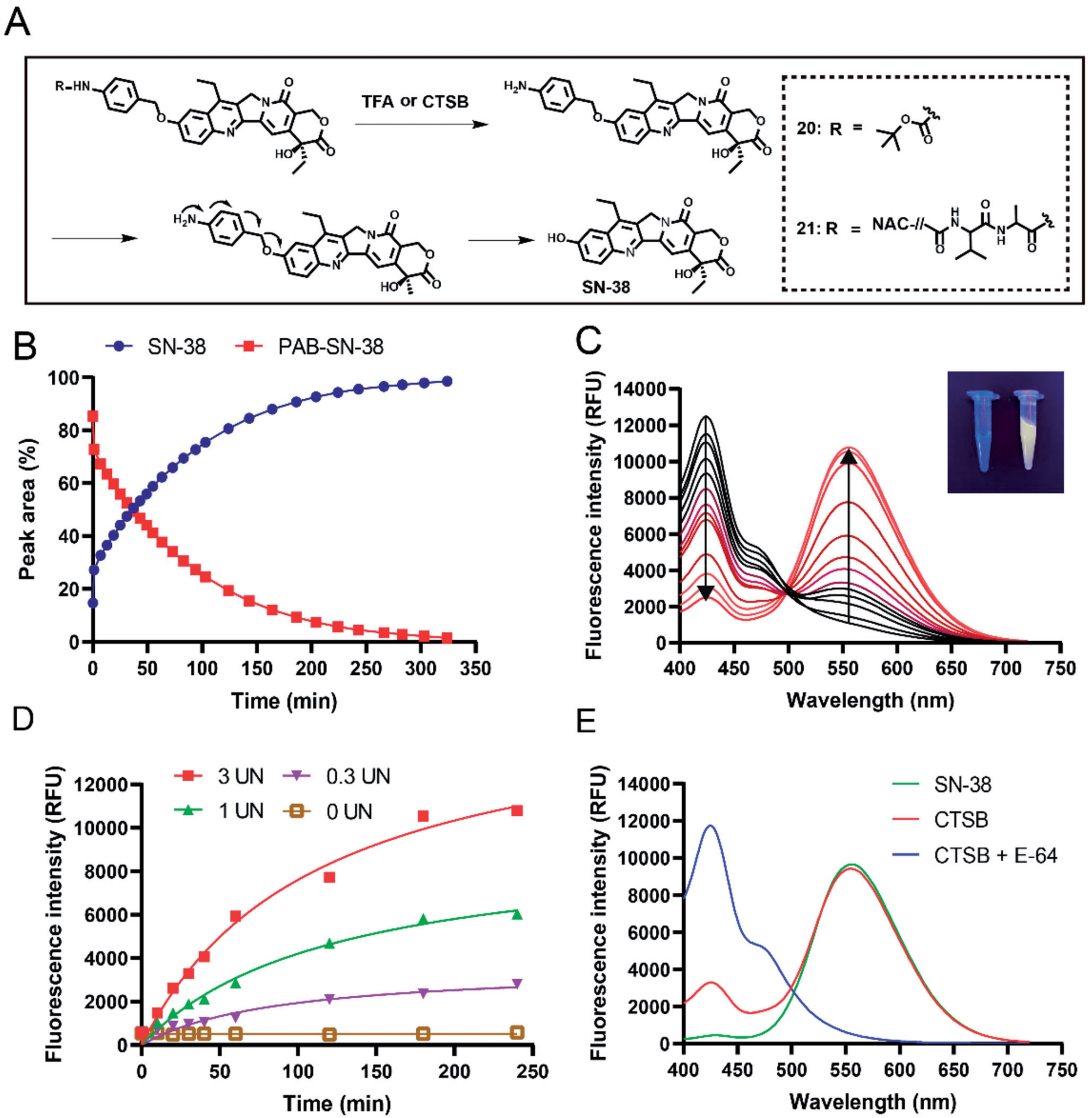
The mechanism of SN-38 release from compound 20, or 21. (A) Schematic illustration of SN-38 release from compound 20, or 21. Compound 20 was triggered by TFA and compound 21 was cleaved by CTSB.NAC: *N*-acetylcysteine. (B) SN-38 release fromPAB-SN-38 was monitored by HPLC in PBS buffer. The monitored wavelength was 380 nM. (C) Fluorescence changes of compound 21 (5 μM) over time (0-4 h) upon exposure to CTSB. (D) Changes in the fluorescence intensity at 550 nm of compound 21 (5 μM) as a function of CTSB concentration. (E) Fluorescence response of compound 21 (5 μM) in the presence of CTSB with or without the CTSB inhibitor E-64.

SN-38 release of compounds 20 or 21 was evaluated at the chemical or CTSB level (Figure 2(A)). Given that PAB-SN-38 is structurally unstable, a Boc protection group was introduced to the amino group of PAB-SN-38 to provide compound 20. Two consecutive steps were employed to determine the SN-38 release from compound 20. First, the Boc protection group was completely removed with trifluoracetic acid. Second, PAB-SN-38 and released SN-38 were monitored at set time points by HPLC after PBS was added to the reaction systems to initiate the self-elimination of PAB. PAB-SN-38 was found to release SN-38 with high efficiency with a half-life t_1/2_ = 36 min (Figure 2(B)), which is far shorter than that of N, N’-dimethyl ethylenediamine spacer of CL2E linker (Govindan et al., 2013). The results demonstrated that CTSB linker direct connection to phenolic hydroxyl group of SN-38 did have a higher efficiency of releasing payloads. Thus, the novel strategy of phenolic ether connection with higher efficiency of releasing payloads may exhibit the potent inhibitory activities comparable to SN-38. At the CTSB level, studies on SN-38 release from compound 21 with CTSB were carried out with the help of the self-fluorescence of SN-38. The fluorescence spectra and UV-Vis absorption of free SN-38 were recorded. An emission maximum was centered at 550 nm, and UV absorption was centered at 373 nm (Figure S1). Initially, the SN-38 present in compound 21 showed an emission maximum at 427 nm. After incubation of compound 21 (5 μM) with 3 UN/mL CTSB, the emission maximum at 550 nm, characteristic of free SN-38 (Shin et al., 2016), gradually increased over time, and the emission band at 427 nm decreased (Figure 2(C)). Under irradiation with light at λ = 365 nm, the physical appearance of the reaction solution observed by the naked eye changed from blue to pale yellow fluorescence (inset Figure 2(C)). Taken together, these findings suggested that free SN-38 could be released from compound 21 in the presence of CTSB. Furthermore, the fluorescence emission of compound 21 at 550 nm was also monitored in the presence of different concentrations (3 UN/mL, 1 UN/mL, 0.3 UN/mL) of CTSB over time. The rate of fluorescence intensity enhancement exhibited dependent on CTSB concentrations (Figure 2(D)). In contrast, no significant fluorescence enhancement at 550 nm was observed after pretreatment with the CTSB inhibitor E-64 (4 μM) (Figure 2(E)). These findings implied that CTSB played a key role in the cleavage of compound 21 and the release of SN-38.

### 3.4. *In vitro* antitumour activity of SN-38-based ADCs with DAR ∼ 4

**Figure 3.**
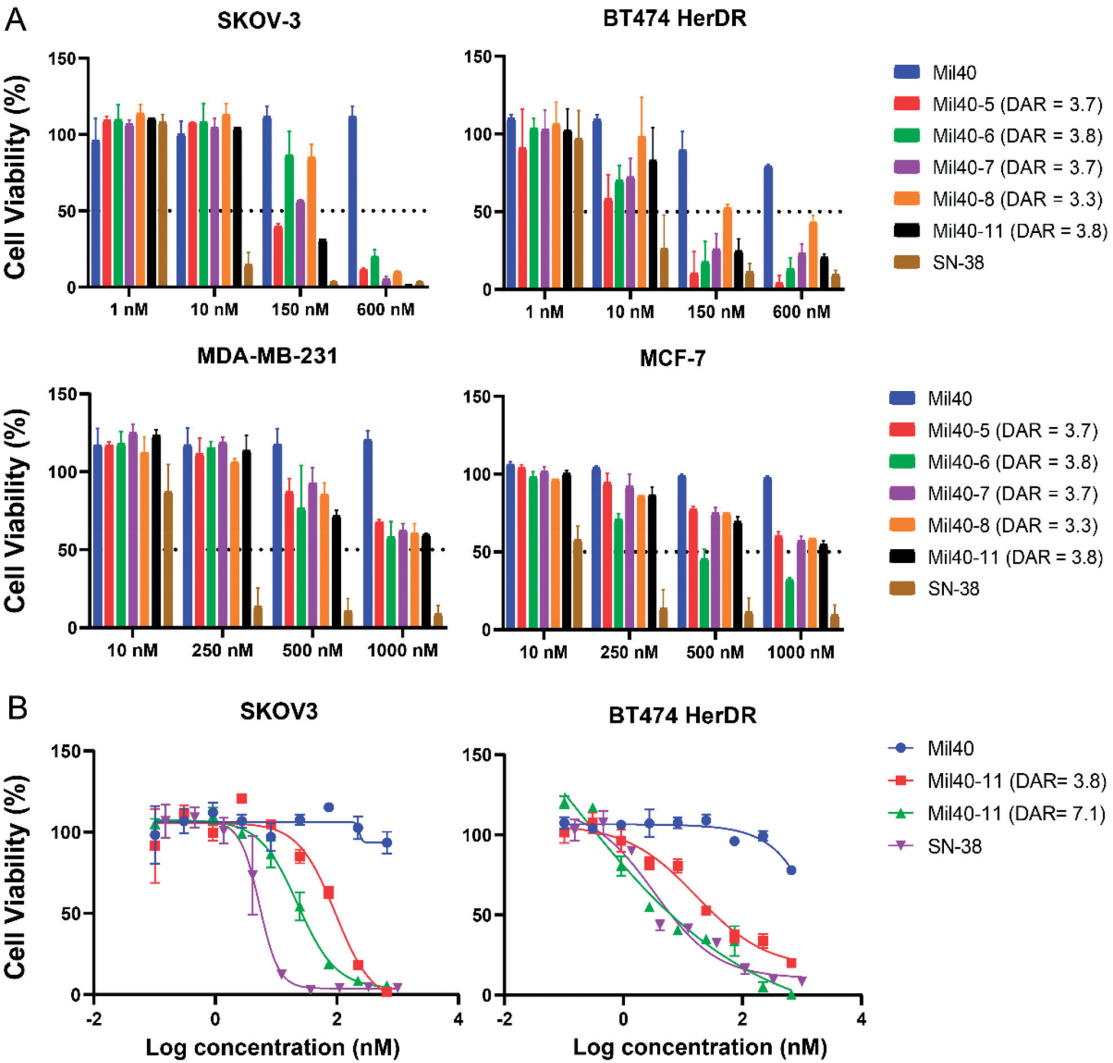
Cytotoxicity of Mil40, SN-38 and ADCs. (A) The antitumour activities of Mil40, SN-38 and ADCs were evaluated in two Her2-positive cells (SKOV-3 and BT474 HerDR) and two Her2-negative cells (MDA-MB-231 and MCF-7). SKOV-3, BT474 HerDR, MDA-MB-231 and MCF-7 cells were cultured under various concentrations of Mil40, SN-38 and ADCs for 10 days, 9 days, 6 days, and 6 days, respectively. Cytotoxicity assays were established using the CellTiter-Go assay kit (CTG) and IC_50_ values were calculated with a 4-variable curve analysis by OriginPro 2018 software. **(B)** Cytotoxicity of Mil40-11 (DAR = 7.1). Cytotoxicity assays of Mil40, SN-38, Mil40-11 (DAR = 3.8), and Mil40-11 (DAR = 7.1) were performed in Herceptin-sensitive SKOV-3 and Herceptin-resistant BT474 cell lines. Data are presented as the mean ± SEM (*n* = 2), and IC_50_ values were calculated from a 4-variable curve analysis by OriginPro 2018.

Her2-positive cell lines SKOV-3 and BT474 HerDR, which are Herceptin-resistant, and Her2-negative cell lines MDA-MB-231 and MCF-7 were selected to evaluate the *in vitro* antitumour activities of SN-38-based ADCs with DAR ∼ 3.7. The Her2 status of these cell lines was previously determined with flow cytometry (Xiao et al., 2021). As shown in Figure 2(A), these ADCs exhibited weak antitumour activities against SKOV-3 and BT474 HerDR cells, with IC_50_ values ranging from 86.3 nM to320.8 nM and from 14.5 nM to 235.6 nM, respectively. For Her2-negative MDA-MB-231 and MCF-7 cells, conjugates did not show antitumour effects, although there was some nonspecific activity associated with higher doses. Mil40 also did not show antitumour activities in selected cell lines regardless of Her2 status, with IC_50_ values greater than 1000 nM. Meanwhile, SN-38 as the payload of ADCs was also evaluated. SN-38 exhibited relatively potent cytotoxicity in the SKOV-3, BT474 HerDR, MDA-MB-231 and MCF-7 cell lines, with IC_50_ values of 10.7 nM, 7.3 nM, 38.9 nM, and 14.4 nM, respectively (Table S1). However, after SN-38 was conjugated to Mil40 to provide ADCs, these conjugates did not show strong cytotoxicity, similar to SN-38, in Her2-positive cell lines. The activities of ADCs were significantly reduced by at least 8 times in SKOV-3 cells and at least 2 times in BT474 HerDR cells (Figure 3 and Table S1). It was speculated that the length of the PEG moiety or the efficiency of releasing SN-38 could influence the antitumour activities of ADCs (Dal Corso et al., 2020). Chemical structure analysis indicated that Mil40-11, a conjugate containing a pendant PEG_8_ moiety, had better antitumour activities than other ADCs, including Mil40-6 with a PEG_2_ moiety, Mil40-7 with a PEG_4_ moiety, and Mil40-8 with a linear PEG_8_ moiety. Notably, Mil40-5 without a PEG moiety also exhibited cytotoxicity similar to Mil40-11. Thus, Mil40-5 and Mil40-11 were preliminarily screened for further studies.

### 3.5. Preparation and characterization of Mil40-11 with DAR = 7.1

Based on the *in vitro* antitumour activities of ADCs with DAR ∼3.7, Mil40-5 and Mil40-11 were further improved for better cytotoxicity. Previous studies indicated that elevating the DAR values would significantly improve the antitumour activities of ADCs, and this strategy was especially common within SN-38-based ADCs (Goldenberg et al., 2015). Thus, for better antitumour activity, ADCs with higher DAR values were obtained by improving conjugation conditions.

**Figure 4.**
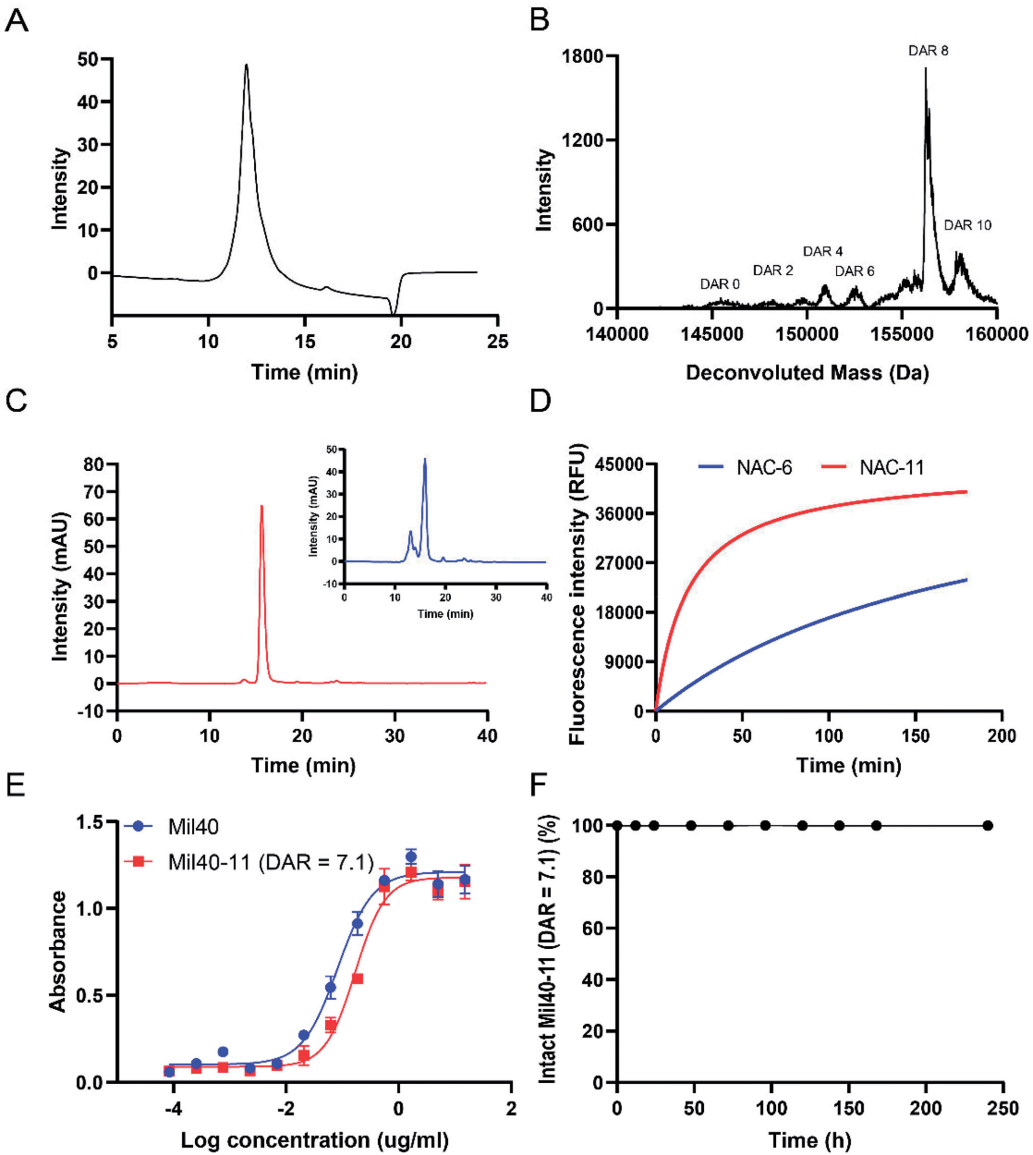
*In vitro* characterization of Mil40-11 (DAR = 7.1). (A) HIC analysis of Mil40-11 (DAR = 7.1). (B) UPLC-Q-TOF-MS analysis of Mil40-11 (DAR = 7.1). (C) Polymerization degree analysis of Mil40-5 (DAR = 6.5) (shown in blue) and mil40-11 (DAR = 7.1) (shown in red). The dimer of the aggregated antibody peak of Mil40-5 was 29%, and that of Mil40-11 was 2%. (D) Changes in the fluorescence intensity of NAC-6 and NAC-11 (10 μM) at 550 nm with the same CTSB concentration. (E) Relative affinities of Mil40 and Mil40-11 (DAR = 7.1) for the antigen protein Her2. (F) Stability analysis of Mil40-11 (DAR = 7.1) in 50% plasma at 37 °C. Data are presented as the mean ± SEM (*n* = 2).

HIC analysis showed that Mil40-11 and Mil40-5 were predominantly composed of species with a DAR value of 8. UPLC-Q-TOF-MS analysis determined that the percentage of DAR8 accounted for 78%, 51%, respectively, which indicated that we achieved the preparation of ADCs with high DAR values (Figure 4(A,B) and Figure S2). The specific DAR values of Mil40-5 (DAR = 6.5) and Mil40-11 (DAR = 7.1) were calculated on the UPLC-Q-TOF-MS analysis. However, according to the size exclusion chromatography (SEC) analysis, Mil40-5 (DAR = 6.5) showed more aggregates with aggregation rate of 29% than that of Mi40-5 (DAR = 3.7) with aggregation rate of 1% while Mil40-11 (DAR = 7.1) had less aggregates with an aggregation rate of 2% (Figure 4(C) and Figure S3). These findings indicated that Mil40-11 had an acceptable low aggregation rate even with a high drug load, which could be attributed to the incorporated hydrophilic pendant PEG_8_ moiety. In contrast, Mil40-5 had an unacceptably high aggregation rate after the DAR value was increased because of its hydrophobicity, which may contribute to its high clearance rate *in vivo* (Lyon et al., 2015). The S + Sw value of compound 11 was 0.008, as predicted by the ADMET predicator 8.5 higher than that of compound 5 (0.005), which indicates that compound 11 may have better water solubility and is more suitable for construction into ADCs with a high drug load. Moreover, with the help of the self-fluorescence of SN-38, compounds 6 and 11 were selected to study the effect of the length of the PEG moiety on the SN-38 release efficiency of linker-SN-38. NAC-11 was found to release SN-38 with higher efficiency than NAC-6 (Figure 4(D)), which implied that a longer PEG moiety seemed beneficial for SN-38 release. Taken together, these findings suggested that Mil40-11 (DAR = 7.1) is worthy of further study.

The effect of increasing DAR values of Mil40-11 (DAR = 7.1) on the binding affinity to Her2 antigen was determined using ELISA. The binding of Mil40-11 (DAR = 7.1) and Mil40 was comparable, with EC_50_ values of 0.17 µg/mL and 0.08 µg/mL, respectively (Figure 4(E)). Thus, these results implied that Mil40-11 (DAR = 7.1) retained a high affinity to Her2 antigen.

The stability of Mil40-11 (DAR = 7.1) was evaluated in diluted human serum using LC-MS/MS analysis. As shown in Figure 4(F), approximately 0.6% (∼2.33 nM) SN-38 released from Mil40-11 was detected after incubation for 10 days in 50% plasma, which indicated that Mil40-11 (DAR = 7.1) was highly stable and that the application of the CTSB cleavable linker used for SN-38-based ADCs met the requirements for the design of ADCs.

The above data demonstrated that the strategy of elevating DAR values was feasible in terms of the construction and quality control of ADCs. Mil40-11 (DAR = 7.1) exhibited significant advantages in reducing the aggregation rate and releasing SN-38 with high efficiency. Thus, Mil40-11 (DAR = 7.1) was selected as the target ADC.

### 3.6. The targeting and internalization of Mil40-11 (DAR = 7.1)

**Figure 5.**
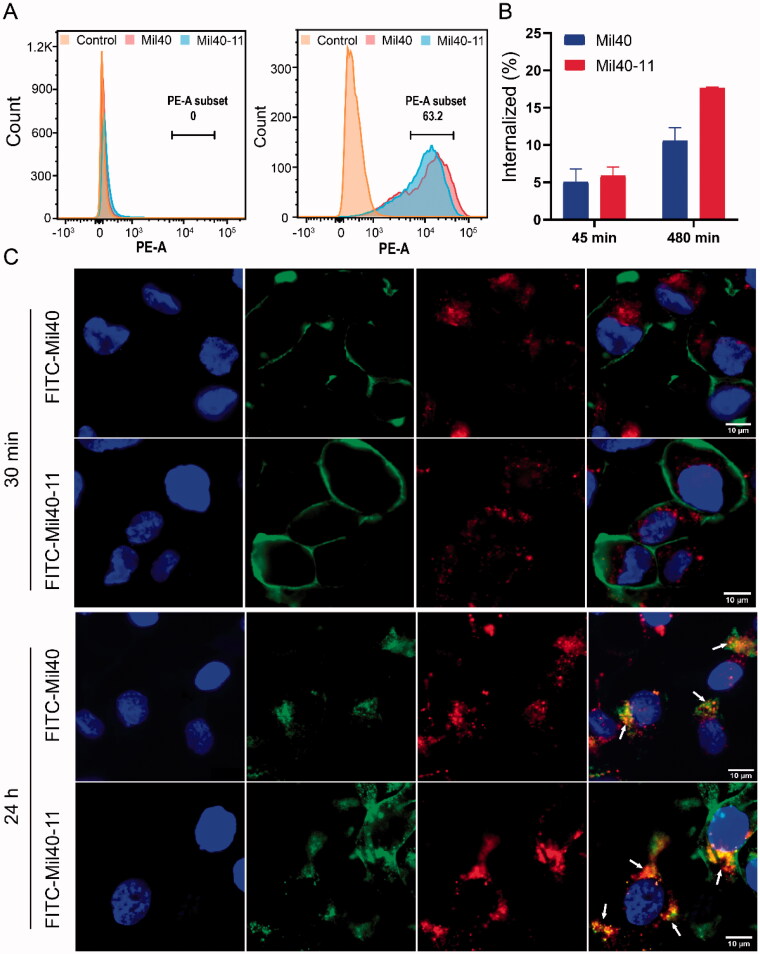
Cell binding and endocytosis of Mil40-11 (DAR = 7.1). (A) Mil40 and Mil40-11 (DAR = 7.1) binding to the Her2 antigens of SKOV-3 and MDA-MB-231 cells. (B) Internalization of Mil40 and Mil40-11 (DAR = 7.1) in SKOV-3 cells. (C) Cell binding and endocytosis of Mil40-11 (DAR = 7.1). Cell binding analysis of Mil40 or Mil40-11 (DAR = 7.1) was observed after incubation with SKOV-3 cell lines at 4 °C for 30 min. The endocytosis ability of Mil40 or Mil40-11 (DAR = 7.1) was observed after incubation with SKOV-3 cell lines at 37 °C for 24 h. Mil40 or Mil40-11 (DAR = 7.1) labeled by FITC is shown in green, Lyso-Tracker red-stained lysosomes are shown in red, and DAPI-stained nuclei are shown in blue. Colocalization signals for Mil40 or Mil40-11 (DAR = 7.1) with lysosome markers are shown yellow. Scale bars: 10 μm.

Flow cytometry was applied to determine the targeting of Mil40-11 against Her2-positive SKOV-3 cell lines and Her2-negative MDA-MB-231 cell lines. As shown in Figure 5(A), Mil40-11 showed similar targeting ability as Mil40. ADC was highly targeted against SKOV-3 cells, with a mean fluorescence intensity (rMFI) value of 42, while Mil40-11 rarely targeted MDA-MB-231 cells, which was consistent with the status of Her2 expression. The above results demonstrated that conjugating compound 11 onto Mil40 did not affect the targeting of Mil40-11 against Her2-positive cell lines.

The internalization of Mil40-11 was also determined in SKOV-3 cells using flow cytometry. Mil40-11 and Mil40 possessed similar internalization efficiencies. After incubation in SKOV-3 cells for 8 h, the internalization percentage of Mil40-11 was 18%, and that of Mil40 was 11% (Figure 5(B)). These findings indicated that conjugating compound 11 onto Mil40 did not affect the internalization of Mil40-11.

Meanwhile, the results of fluorescence microscopy showed that SKOV-3 cells incubated with FITC-stained Mil40 or FITC-stained Mil40-11 at 4 °C for 30 min showed a similar pattern, where they bound to the Her2 antigens expressed on the cell membrane (shown in green). After incubation at 37 °C for 24 h, the green signal appeared within cells. Through the intracellular signal colocalization, lysosome sections were found to display yellow, which indicated that Mil40-11 was internalized and transported into lysosomes (Kalim et al., 2017) (Figure 5(C)).

### 3.7. *In vitro* antitumour activity of Mil40-11 (DAR = 7.1)

As shown in Figure 2(B), the antitumour activity of Mil40-11 (DAR = 7.1) was significantly enhanced in SKOV-3 cell lines compared with Mil40-11 (DAR = 3.8), with IC_50_ values of 22.2 nM and 86.3 nM, respectively. Moreover, Mil40-11 (DAR = 7.1) exhibited comparable cytotoxicity to SN-38 in BT474 HerDR cell lines, with IC_50_ values of 5.5 nM and 7.3 nM, respectively. Mil40-11 (DAR = 3.8) showed relatively weak antitumour activity with an IC_50_ value of 28.8 nM. Mil40 did not show cytotoxicity in SKOV-3 and BT474 HerDR cell lines. Based on the above data, Mil40-11 (DAR = 7.1) exhibited strong cytotoxicity in Her2-positive cell lines, and its enhanced activity demonstrated that elevating the DAR values would significantly improve the antitumour activities of ADCs. This is observed in this case for *in vitro* antitumor activity and it is observed with compound 11 derived ADCs.

### 3.8. *In vivo* antitumour activities of Mil40-11 (DAR = 7.1)

**Figure 6.**
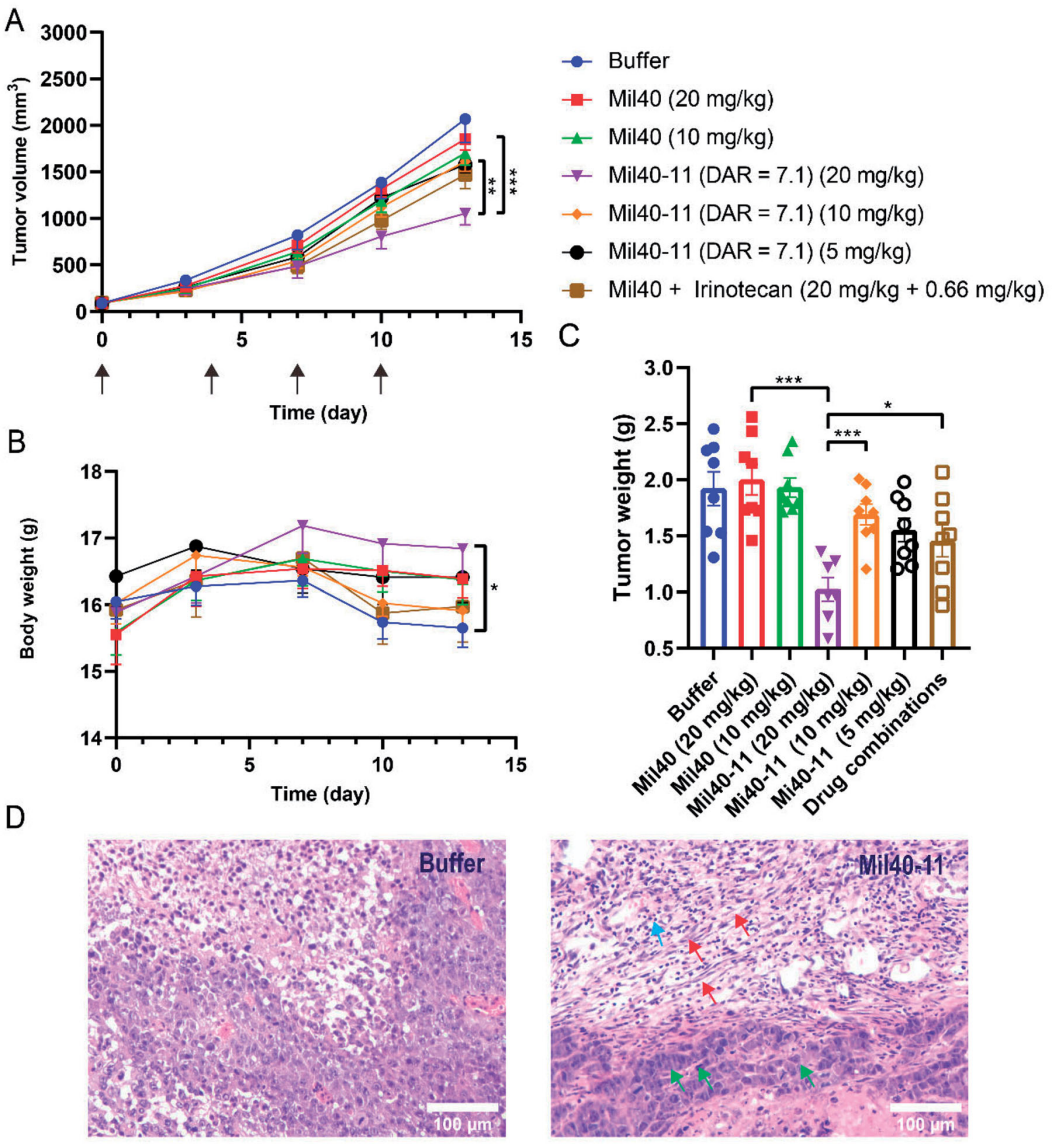
*In vivo* efficacy and tolerability of Mil40-11 (DAR = 7.1) in human tumor xenograft models. (A) Efficacy in the human ovarian cancer model SKOV-3. BALB/c-nude mice were implanted subcutaneously with SKOV-3 tumor tissues. When the size of tumors reached ∼100 mm^3^, tumor-bearing mice were treated with vehicle, Mil40, and Mil40-11 (DAR = 7.1) on days 0, 3, 7 and 10, respectively. The results are shown as the mean ± SEM, *n* = 7–8/group. To compare the difference in activity between Mil40 and Mil40-11 (DAR = 7.1), unpaired two-tailed t tests were used, and statistical analysis was performed using GraphPad Prism 8.0. 2; ** *p* < .005 and *** *p* < .0005. (B) The body weight of mice did not show significant changes during treatment in the subcutaneous xenograft model. Statistical analysis of the difference in body weight was also performed using GraphPad Prism 8.0. 2; *p < .05. (C) Tumor weight of mice in the subcutaneous xenograft model. Statistical analysis of the difference in body weight was also performed using GraphPad Prism 8.0. 2; *p < .05. (D) Histological sections of SKOV3 tumor tissues with H&E staining. Red arrows: fibrosis of tumor. Blue arrows: nucleosome pyknosis. Green arrows: hyperchromatism. Scale bars: 100 μm.

To evaluate the antitumour activities of Mi40-11 (DAR = 7.1), subcutaneous tumor-bearing models of SKOV-3 in female BALB/c nude mice were established. When the tumor volumes grew to approximately 100 mm^3^, the mice were intravenously administered Mil40-11 (DAR = 7.1) (5 mg/kg, 10 mg/kg and 20 mg/kg), Mil40 (10 mg/kg and 20 mg/kg) and the drug combination (20 mg/kg Mil40-11 + 0.66 mg/kg irinotecan) on days 0, 3, 7 and 10. Compared with the 20 mg/kg Mil40 treatment group, the 20 mg/kg ADC treatment group could significantly delay the tumor growth (*p* < .05). The 20 mg/kg ADC treatment group had better antitumour effects, although no significant tumor suppression (*P* > 0.05) was observed compared with the drug combination treatment group (Figure 6(A)).

During the whole treatment period, the body weights of mice treated with 20 mg/kg ADC were significantly higher than those of mice treated with buffer, which implied that ADC could improve the physical condition of tumor-bearing mice to avoid weight loss (Figure 6(B)). In addition, the tumor weight of the 20 mg/kg ADC treatment group was lower than that of the Mil40 group with the same dose and drug combination group (Figure 6(C)). H&E staining showed clear fibrosis of tumor tissue, nucleosome pyknosis and hyperchromatism of tumor cells in the ADC treatment group (Figure 6(D)).

## 4. Conclusions

We reported a novel SN-38-based ADC, Mil40-11, with a high DAR value of 7.1, the linker-drug part of which was constructed by directly connecting the phenolic hydroxyl group of SN-38 to the CTSB cleavable linker through ether bonds. In this study, the feasibility of directly connecting SN-38 to a VA-based linker through the ether bond was determined. With the advantages of the self-fluorescence of SN-38, the high SN-38 release efficiency of compound 21 was observed in the presence of CTSB. Upon exposure to CTSB, the emission maximum of compound 21 quickly shifted from 427 nM to 550 nM, characteristic of SN-38. After CTSB was pretreated with the CTSB inhibitor E-64, no emission maximum was observed. Thus, it was concluded that an ether bond connection could quickly release SN-38 after self-elimination of PAB and CTSB played a key role in this process.

We evaluated the antitumour activities of ADCs with DAR ∼ 3.7 in a series of Her2-positive tumor cells. Compared with SN-38 cells, ADCs displayed significantly lower activities in the SKOV-3 and BT474 cell lines. The strategy of elevating the DAR values of ADCs, especially common in SN-38-based ADCs, was adopted for better antitumour effects. Consequently, the increased hydrophilicity of compound 11 enabled Mil40-11 to be prepared with a high DAR value of 7.1 without a significant aggregation rate (2%), while the aggregation rate of compound 5-based ADCs with a DAR value of 6.5 reached 29% because of its hydrophobicity. In addition, the length of the PEG moiety could influence the efficiency of linker-SN-38 in the presence of CTSB. NAC-compound 11 with a pendant PEG_8_ moiety released SN-38 faster than NAC-compound 6. Thus, Mil40-11 (DAR = 7.1) was finally screened for further research.

Compared with Mil40, the targeting and internalization of Mil40-11 (DAR = 7.1) was not affected during conjugation. Its stability in human serum demonstrated that the adopted phenolic ether connection addressed the instability of SN-38-based ADCs and could be incorporated into the design of ADCs. Furthermore, elevating the DAR values enhanced the cytotoxicity of Mil40-11, which exhibited *in vitro* antitumour activities comparable to SN-38 and significantly delayed tumor growth.

In summary, the ether bond incorporated into SN-38-based ADCs displayed high efficiency of releasing SN-38, stability in human serum and acceptable antitumour activities. These exciting findings demonstrated that the strategy of ether bond connection broadens the applications of SN-38 in ADCs and further provides another choice for CTSB cleavable triggers connecting other hydroxyl-bearing toxins.
